# Isolated Hodgkin lymphoma of the intracranial dura: A case report and review of the literature

**DOI:** 10.1002/ccr3.7562

**Published:** 2023-06-22

**Authors:** Tharsan Kanagalingam, Vikram Velker, Shervin Pejhan, Qi Zhang, Joy Mangel, Sympascho Young

**Affiliations:** ^1^ Schulich School of Medicine and Dentistry University of Western Ontario London Ontario Canada; ^2^ Department of Radiation Oncology, London Regional Cancer Program London Health Sciences Center London Ontario Canada; ^3^ Department of Neuropathology London Health Sciences Center London Ontario Canada; ^4^ Division of Hematology, Department of Medicine London Health Sciences Center London Ontario Canada

**Keywords:** central nervous system, Hodgkin lymphoma, involved site radiotherapy, primary dural lymphoma

## Abstract

Primary dural Hodgkin lymphoma (PDHL) is an extremely rare subset of Hodgkin lymphoma (HL). Its existence is controversial, as Hodgkin lymphoma is not traditionally thought to arise from the central nervous system (CNS) or its meninges and only 0.02% of patients with Hodgkin lymphoma have any CNS involvement. We report a case of a 71‐year‐old Caucasian man who presented with progressive fatigue and sudden onset slurred speech, disorientation, and memory loss. Brain imaging identified a large extra‐axial right frontal mass, and he underwent urgent subtotal resection. Pathology and subsequent workup revealed Stage IAE classical Hodgkin lymphoma of the right frontal dura, with no extra‐cranial disease or leptomeningeal spread detected. The patient was subsequently treated with ABVD chemotherapy (completed 2.5 of 4 planned cycles) and 36 Gy in 20 fractions of consolidative involved‐site radiotherapy (ISRT). He has been followed for 5 years with no clinical or radiological signs of recurrence. This is the second confirmed case of intracranial PDHL reported in the literature, with the longest follow‐up for any case of PDHL.

## Graphical abstract

Rare case of a 71 year old man with intracranial primary dural Hodgkin lymphoma (PDHL). Patient underwent subtotal resection of the mass, followed by adjuvant chemotherapy and consolidative radiotherapy, with no recurrence at 5 years follow up.

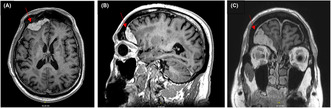

## 1 INTRODUCTION

Malignant lymphomas account for the third most common neoplasm in the head and neck region. Non‐Hodgkin lymphoma (NHL) is more common than Hodgkin lymphoma (HL) and typically arises in lymph nodes anywhere in the body, whereas HL typically occurs in the upper body. Central nervous system (CNS) involvement for patients with Hodgkin lymphoma is rare, and more commonly a result of secondary disease processes. It is estimated that only 0.02% of patients with Hodgkin lymphoma have CNS involvement.^1^ Thus, Hodgkin lymphoma confined to the CNS represents a very rare subset of the disease. Primary dural lymphoma (PDL) is a rare subset of primary central nervous system lymphoma (PCNSL) that is confined to the dura mater. Reports of PDL in the literature have mainly been subtypes of non‐Hodgkin lymphoma (NHL).^2^ A retrospective analysis of 316 cases of PCNSL (all NHL) at a single institution included 20 cases (6.3%) of PDL.^3^ In contrast, to date, there are only four published cases of PDHL^4^, ^5^, ^6^, ^7^ and only one was an intracranial PDHL, while the rest were cases of spinal PDHL. Despite the rarity and the absence of guidelines, PDL of both NHL and HL subtypes have a better prognosis with overall and remission‐free survival relative to cases of systemic disease with secondary CNS involvement.^3^

On initial presentation, PDHL patients are often symptomatic due to tumor mass effects, commonly presenting with headaches, cranial nerve deficits, dysesthesias, vertigo, and/or confusion. Due to the lack of specific HL‐related symptoms, the initial diagnosis of PDHL can be challenging as it often mimics meningioma on imaging, and histopathological confirmation typically requires surgical intervention for tissue sampling or excision. Depending on the extent of CNS involvement, attempts at complete excision and subsequent therapeutic management decisions can be challenging.^8^, ^9^

In this report, we describe the case of a 71‐year‐old Caucasian male with primary‐stage IAE Hodgkin lymphoma of the right frontal dura who was treated with subtotal resection, ABVD chemotherapy, and consolidative involved‐site radiotherapy (ISRT). He has been followed for 5 years with no signs of recurrence. This is the second case of intracranial PDHL reported in the English literature.

## 2 CASE REPORT

In October 2016, a 71‐year‐old Caucasian man presented to a community emergency department in Ontario with a likely episode of unwitnessed seizure, new‐onset slurred speech, disorientation, memory loss, and lightheadedness. He also had a one‐week history of profound progressive fatigue. He did not have a measured fever, drenching night sweats, or weight loss. His past medical history included dyslipidemia, hypertension, obstructive sleep apnea, chronic lower back pain, and a previous lacunar stroke from which he had full recovery of function. The patient was managed initially in the emergency department with low‐dose aspirin, dexamethasone, and phenytoin.

**FIGURE 1:**
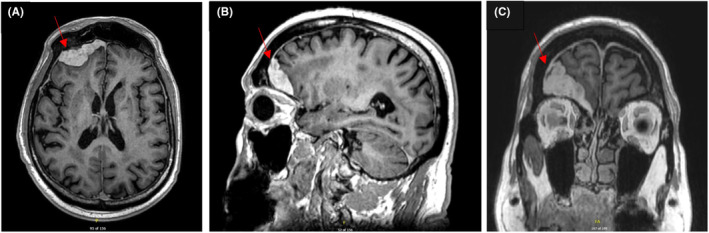
Oct 17, 2016 (Pre–op). MRI head, post gadolinium 3‐D T1 axial and sagittal of primary dural Hodgkin lymphoma. Right frontal dural mass visualized with vasogenic edema in the right frontal white matter.

**FIGURE 2:**
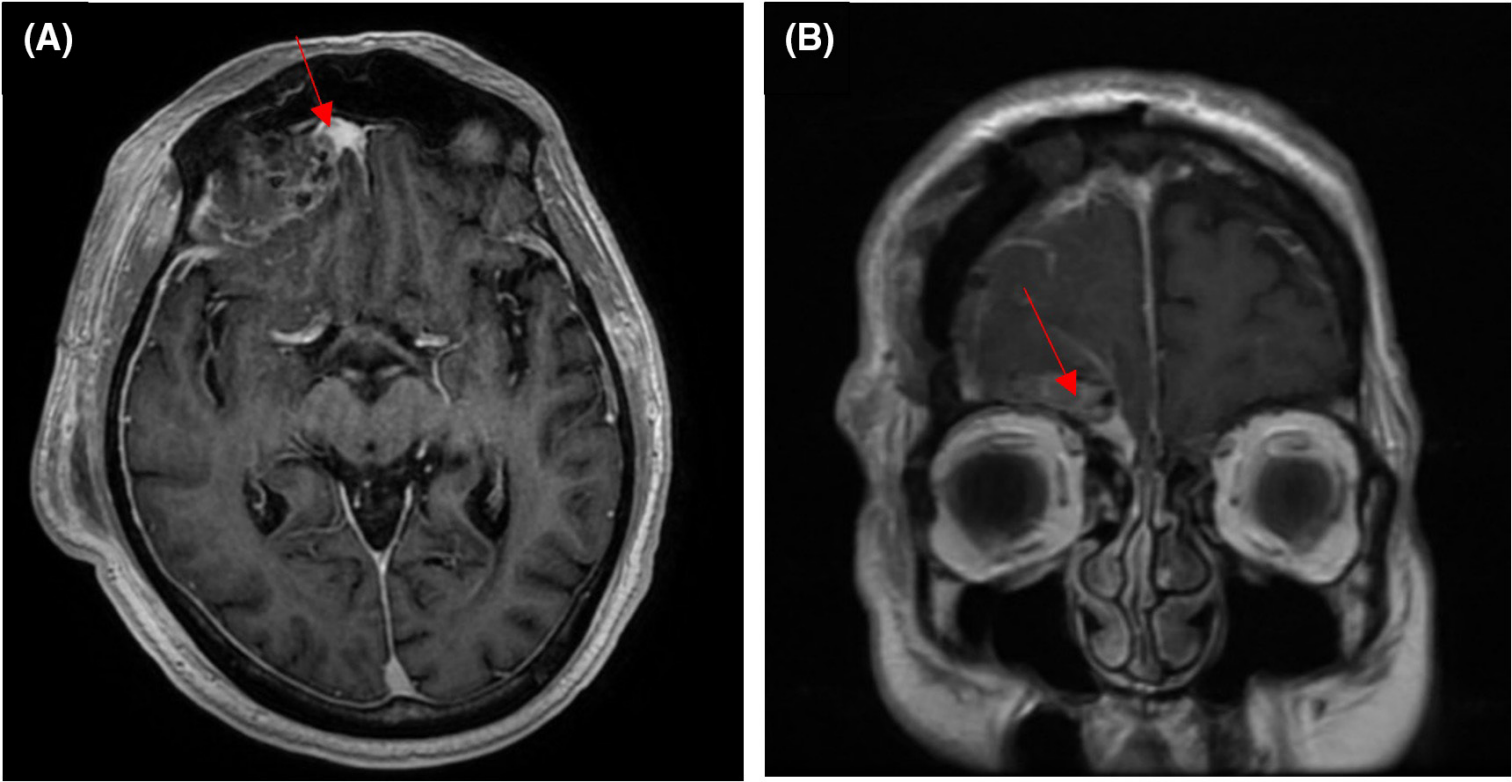
Oct 19, 2016 (Day 1 post–op). MRI head post gadolinium T2 SSFSE axial and sagittal views, showing irregular resection cavity with 2.7 × 0.9 cm band enhancing tissue along the medial supraorbital margin appears to be a residual tumor.

CT head with contrast revealed a 5 × 3.5 cm extra‐axial mass with smoothly marginated borders, overlying the right frontal lobe in the anterior cranial fossa. There was a mass effect on the right frontal lobe with a moderate degree of surrounding vasogenic edema. The initial radiologic impression was likely meningioma based on the round appearance and wide base on the floor of the dura at the right frontal fossa, along with a homogenous pattern of enhancement. MRI brain completed the subsequent day confirmed the tumor, but raised suspicions of lymphoma, as the mass had reduced in size in response to dexamethasone (Figure 1). The patient was referred urgently to neurosurgery and underwent a same‐day right frontal craniotomy with a duraplasty laid down over the defect in the orbital dura using pericranium. Maximal safe resection was done (estimated resection of >90% of the tumor), but some residual tumor by the cribriform plate was left to avoid permanent CSF leakage. Subsequent CT chest/abdomen/pelvis did not find any evidence of extra‐cranial disease, though a large left‐lower lobe pulmonary embolism was found and treated. MRI completed on postoperative day 1 revealed the post‐resection cavity and a 2.7 cm band of enhancing tissue along the medial supraorbital margin felt to be residual tumor (Figure 2). A post‐op CSF leak later developed, requiring the insertion of a lumbar drain on post‐op day 9.

**FIGURE 3:**
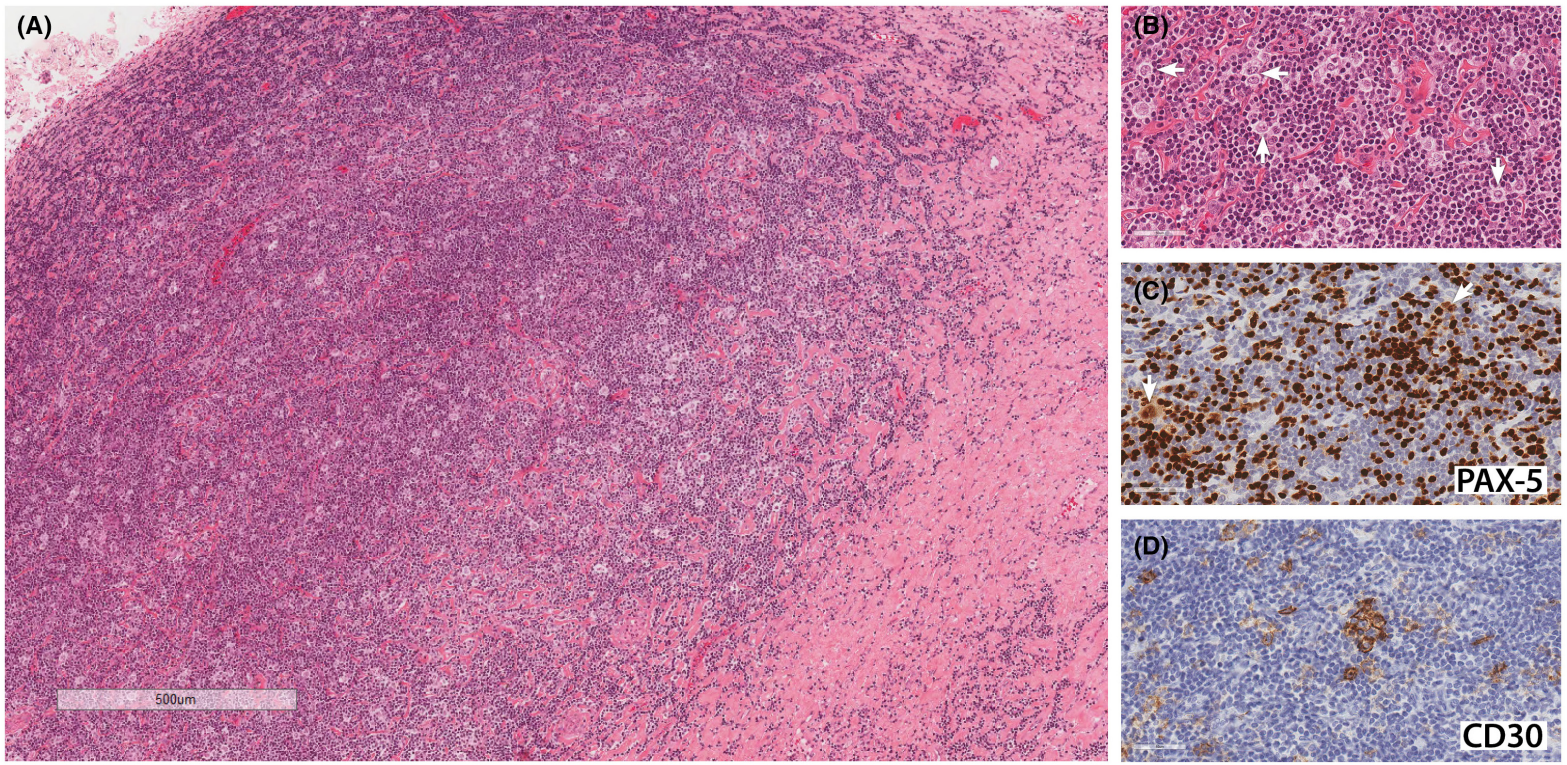
Pathology slides. (A) Larger atypical Hodgkin cells with pale cytoplasm are scattered in a background of smaller lymphocytes and fibrotic tissue (H&E stain), 40×. (B) Hodgkin cells have vesicular nuclei with prominent nucleoli (H&E stain), 400×. (C) These cells show weaker positivity for Pax‐5 (white arrows), compared to the smaller reactive B lymphocytes in the background, 400×. (D) CD30 shows a membranous and para nuclear Golgi region immunoreactivity in Hodgkin cells, 400×.

Microscopic examination showed a lymphoproliferative neoplasm characterized by extensive areas of fibrosis, collections of small lymphocytes, and scattered larger atypical Hodgkin cells with pale eosinophilic cytoplasm, vesicular nuclei, and prominent nucleoli (Figure 3A). The latter cells were immunoreactive for CD30 and co‐expressed PAX‐5 in a weaker manner compared to the background reactive B lymphocytes (Figure 3C,D). These histological features are consistent with classical Hodgkin's lymphoma.

Blood work showed leukocytes 12.3 × 10^9^, erythrocytes 5.13 × 10,^9^ thrombocytes 340 × 10,^9^ neutrophils 8.6 × 10,^9^ lymphocytes 2.1 × 10,^9^ monocytes 1.4 × 10,^9^ eosinophil 0.1 × 10,^9^ basophil 0. Serology was negative for Hepatitis B, C, and HIV. Cardiac left ventricular ejection fraction was 74% on echocardiogram. Bone marrow aspirate and biopsy were negative for lymphoma. Lumbar puncture did not identify any leptomeningeal involvement. A full‐body PET/CT scan showed no metabolic activity at the resection site and no extracranial sites of disease. MRI revealed the residual disease following sub‐total resection. The final diagnosis was Stage IAE classical Hodgkin lymphoma involving the right dura.

**FIGURE 4:**
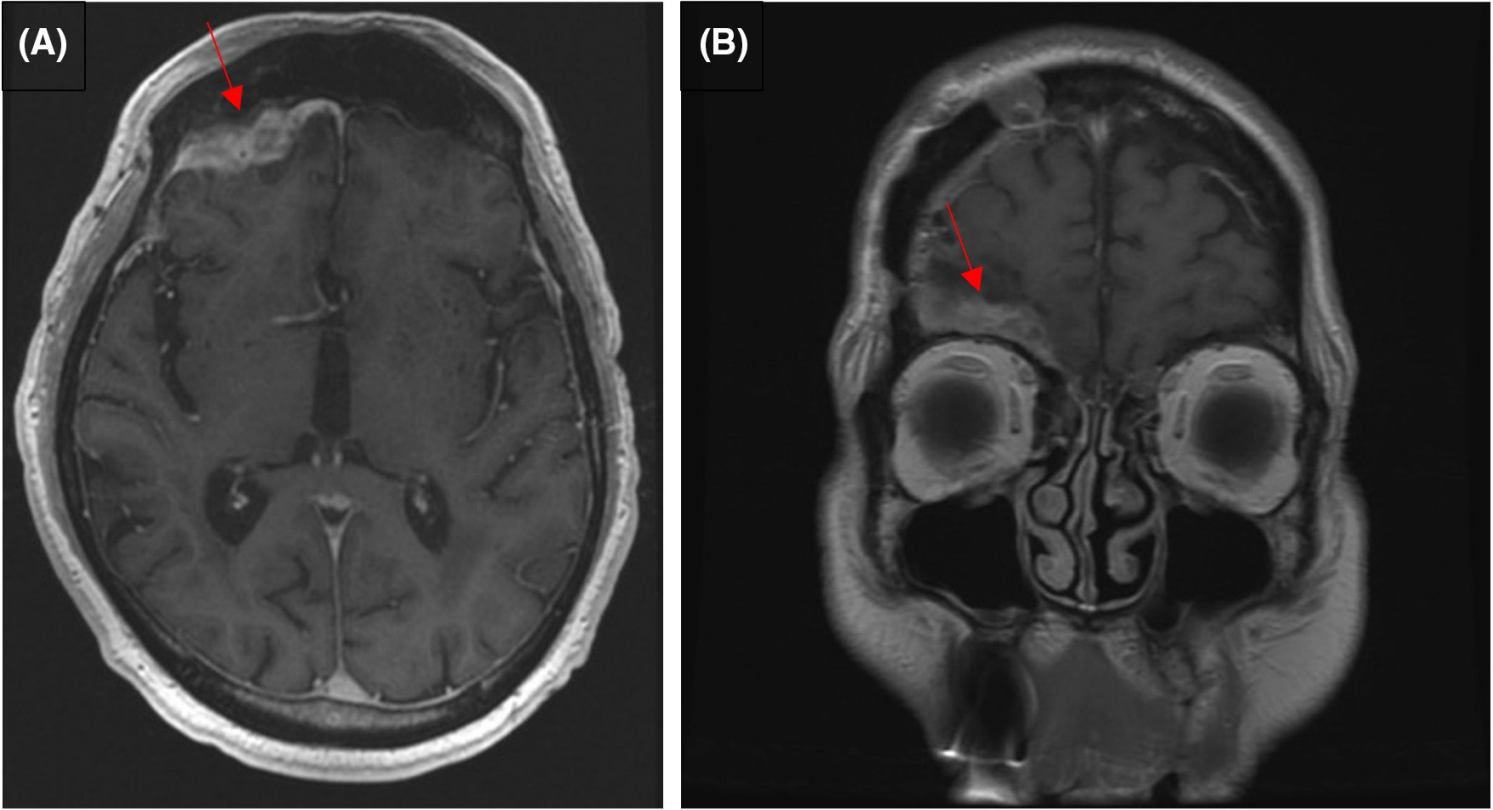
March 19, 2017 (1 month post–chemotherapy). MRI head post gadolinium 3D T1 axial and sagittal views showing thick lobulated enhancement of the dural margin increasing in size to 4.5  × 1.4 cm, felt to represent residual tumor mixed with reactive dural enhancement.

The case was reviewed at a multidisciplinary case conference and a recommendation was made to treat the patient with four cycles of adriamycin, bleomycin, vinblastine, and dacarbazine (ABVD) followed by ISRT. The patient started cycle IA on Nov 18, 2016. Unfortunately, his treatment was complicated by febrile neutropenia requiring hospital admission and IV antibiotics, which resulted in a dose reduction of cycles 1B and 2A. The patient completed cycle 3A of ABVD chemotherapy, but chemotherapy was then discontinued at the patient's request due to poor quality of life from intolerable side effects of general malaise and fatigue. A post‐chemotherapy MRI head revealed an improving cystic collection at the previous surgical site, however the thick lobulated enhancement of the dural margin had increased in size, to 4.5  × 1.4 cm, felt to represent residual tumor mixed with reactive dural enhancement (Figure 4).

After 4 weeks post‐chemotherapy, the patient went on to receive a 4‐week course of consolidative ISRT (3600 cGy in 20 fractions) to the right frontal region of the brain. Radiation volumes were delineated using an involved site definition focusing on the right dura post‐operative tumor bed plus residual fibrosis/tumor tissue. Radiation was delivered via Linac‐based intensity modulated volumetric arc technique (IMRT‐VMAT), using two opposing arcs and 6 mV photons. He did not receive any dexamethasone during RT. There were no unanticipated or severe acute or subacute toxicities observed.

**FIGURE 5:**
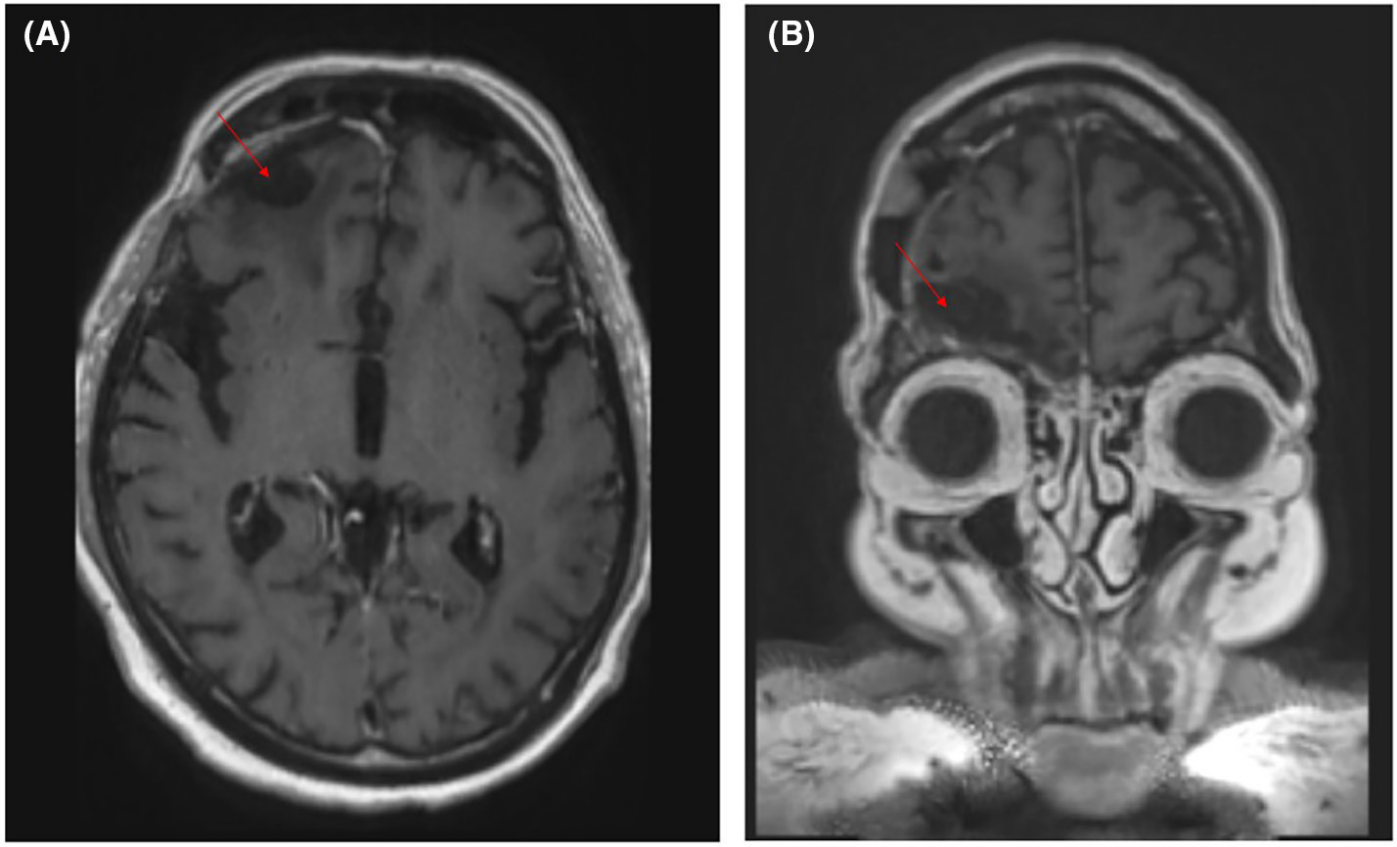
January 18, 2019 (2 years post–chemotherapy and radiation). MRI head, post gadolinium 3D T1 axial and sagittal views showing no evidence of recurrence in the brain.

Following the completion of radiation, the patient underwent a surveillance CT head every 3 months for the first year. He was discharged from routine follow‐up at the cancer center in Feb 2019 at his request. Subsequent clinical follow‐up care was provided by his primary care provider, and this consisted of yearly visits and blood work (including CBC and LDH). The patient continues to be stable neurologically and is followed by a neurologist for antiepileptic management, on an annual basis, with no breakthrough seizure activity in over a year. Follow‐up imaging with MRI head in Jan 2019 (Figure 5) and CT head in Nov 2021 (not shown) were both negative for progression. He is now over 5 years from initial diagnosis with no clinical or radiological signs of recurrence, representing a high likelihood of cure.

## 3 DISCUSSION

The vast majority of PDL cases are categorized as NHL, so clinical information is scarce regarding PDHL^10^. There have only been four published case studies of PDHL,^4^, ^5^, ^6^, ^7^ three cases of extracranial PDHL, and one case of an intracranial PDHL, like in our patient (Table 1). To our knowledge, this is only the second reported case of intracranial PDHL, and it was successfully managed with subtotal resection, chemotherapy, and ISRT.

**TABLE 1:** Case reports of PDHL.

Year	Author	Age	Sex	Location	Surgical resection	Chemotherapy	Radiation therapy	Outcome and follow up
1980	Nagashima et al.^4^	60	M	Falx cerebri	Sub‐total resection	2 cycles of vincristine, prednisolone, nimustine, and procarbazine	52 Gy to the whole brain	NED at 13 months. Death due to pneumonia/meningitis
2006	Heran et al.^6^	42	M	Intradural extramedullary cervical region	None	6 cycles ABVD	None	NED at 48 months
2010	Chotai et al.^5^	24	M	Intradural extramedullary region	None	3 cycles ABVD	None	Stable disease at 12 months
2020	Williamson et al.^7^	48	F	Intradural of conus medullaris and cauda equina	Sub‐total resection	None	RT 36 Gy in 20 fractions to T10‐sacrum	NED at 10 months

Abbreviation: NED, no evidence of disease.

Our patient presented with slurred speech, memory loss, and lightheadedness, due to tumor mass effects, in contrast to published cases of PDHL that presented with neuromuscular weakness due to intradural spinal masses.^5^, ^6^, ^7^ The patient was initially managed with dexamethasone, prior to neurosurgery which reduced the size of the tumor. The role of upfront and extensive surgery is not fully delineated in PDL. In the setting of NHL, maximal resection results in improved local control, and a retrospective analysis identified the extent of surgical resection as a positive prognostic marker for overall survival.^3^ In our case, the patient underwent a near‐total resection, however, some residual tumor was left in situ due to its proximity to the cribriform plate. In previous case reports (Table 1), surgical intervention was completed in 50% of the cases. Surgical resection of tumors was initially planned in the other two cases but was aborted when extensive spinal involvement of the mass was identified following laminectomy. In cases without surgical intervention, chemotherapy was the sole therapy. Chemotherapy was given in 75% of the reported cases. In our case, 2.5 of the four planned cycles of ABVD were delivered systemically, without any intrathecal chemotherapy. The abbreviated course resulted in a partial response, with reduction but not complete resolution of the residual enhancing tissue.

In the extracranial setting, Hodgkin lymphoma is very responsive to both chemotherapy and radiation. Since the ABVD chemotherapy regimen was first introduced in 1975, it has been the standard of care for first‐line treatment of classical Hodgkin lymphoma without CNS involvement.^11^ Treatment options for limited‐stage HL include either combined modality therapy (abbreviated chemotherapy followed by radiotherapy) or chemotherapy alone. Owing to the potential long‐term toxicities of radiotherapy, the most recent therapeutic paradigms have sought to reduce radiation volumes and doses, and even to eliminate radiation by using PET‐directed strategies.^12^, ^13^, ^14^

In this clinical case, following chemotherapy, ISRT, a modern approach to radiation, was employed successfully. Thirty–six Gy was chosen given that the patient did not complete the 4 planned cycles of chemotherapy, and there was also radiologic evidence of residual dural enhancement post‐chemotherapy (Figure 1C,D). The volume of radiation included the entire post‐operative bed including areas of dural enhancement, as delineated by the pre‐chemotherapy MRI head. This dose was in line with the successful management of a case of stage IE lumbosacral spinal PDHL by Williamson et al, using 36Gy in 20 fractions for residual tumor following subtotal resection. Their patient received radiation alone, with no systemic chemotherapy, and remained in remission 10 months post‐treatment.^7^ Although the follow‐up duration was short, this raises the possibility that chemotherapy could potentially be omitted altogether in a setting like this.

Due to the favorable prognosis of early‐stage Hodgkin lymphoma, there must be a careful balance between providing sufficient therapy to eradicate the disease while also minimizing long‐term sequelae from treatment.^15^ This is especially true for radiation with its late effects on normal tissue, with careful attention to adequate tumor coverage and dose while sparing surrounding healthy tissue. Standard ISRT doses for extracranial HL range from 20 to 30 Gy depending on the histology, clinical presentation, and the presence or absence of suspected residual disease following chemotherapy.^16^ The optimal chemotherapy regimen for PDHL is unknown. ABVD is thought to have poor penetration of the blood–brain barrier (BBB) but given the positive outcomes in patients with primary dural MALT lymphoma treated with systemic chemotherapy without CNS penetration, the dura may not be protected by the BBB as previously thought^17^ Age and frailty also need to be considered when choosing a chemotherapy regimen.

To make an early diagnosis of PDHL or any PCNSL subtype, the disease must be limited to the CNS; this contrasts with secondary CNS involvement by systemic lymphoma. The international PCNSL Collaborative Group recommends screening for extra‐cranial involvement using a CT scan of the chest, abdomen, and pelvis, whole body fluorodeoxyglucose positron emission tomography (FDG‐PET), bone marrow biopsy, and testicular ultrasound (in males). Recent studies have questioned the necessity of bone marrow biopsy in the presence of a negative PET scan. A retrospective study found that only 0.6% (2/352) of patients with presumed PCNSL with negative PET scans had bone marrow biopsy findings that impacted management.^18^

A limitation of the current landscape of PDHL case reports is the lack of long‐term follow‐up data, with a median follow‐up of 12 months (range, 10–48 months) post‐adjuvant therapy after completion of chemotherapy or radiation. (Table 1). The optimal surveillance strategy and duration for patients with PDHL are unknown and are typically extrapolated from routine practices for extracranial presentations of HL. Recurrences for early‐stage HL are most common within the first 2 years following treatment and are exceedingly rare after 5 years, so ongoing follow‐up at a cancer center beyond that point is generally not required.

## 4 CONCLUSION

In summary, we describe a case of primary HL of the intracranial dura. To our knowledge, there has only been one previous case reported in the literature. Due to its rarity, the natural history of the disease and optimal treatment strategies are not well understood. This case of PDHL with 5 years of follow‐up demonstrated a favorable outcome after partial surgical resection followed by ABVD chemotherapy and ISRT. More extensive experience with PDHL is needed to provide further insight into the prognosis and optimal management of this rare condition.

## AUTHOR CONTRIBUTIONS

**Tharsan Kanagalingam:** Investigation; writing – original draft; writing – review and editing. **Vikram Velker:** Conceptualization; funding acquisition; investigation; supervision; writing – review and editing. **Shervin Pejhan:** Writing – original draft. **Qi Zhang:** Supervision. **Joy Mangel:** Supervision; writing – review and editing. **Sympascho Young:** Supervision; writing – original draft; writing – review and editing.

## FUNDING INFORMATION

None.

## Data Availability

None.
